# Vaccination against Yellow Fever

**Published:** 1885-03

**Authors:** 


### VACCINATION AGAINST YELLOW FEVER.

The researches which have during the past two years been made by Dr. Domingos Friere have now reached a new point of departure. This investigator has prepared an attenuated virus with which he proposes to vaccinate individuals, with a view to rendering them proof against the occurrence of yellow fever. The Emperor of Brazil, having regard to the alleged innocuousness of the prepared virus, has authorized the practice of vaccination. Dr. Friere has accordingly vaccinated five hundred individuals. Three captains and all the crews of English vessels have been vaccinated with a view of escaping the infection of yellow fever, which prevails at Rio Janerio. Thus far none of the vaccinated have been attacked by the disease, and none of them suffered the least inconvenience from the operation. M. Bouley, who gave the facts to the Academie de Medicine, while implicitly believing the above narrated facts, does not yet implicitly accept the views of Dr. Friere on the Micrococcus xanthogenicus.—*Lancet.*

